# Spontaneous Altruism by Chimpanzees and Young Children

**DOI:** 10.1371/journal.pbio.0050184

**Published:** 2007-06-26

**Authors:** Felix Warneken, Brian Hare, Alicia P Melis, Daniel Hanus, Michael Tomasello

**Affiliations:** Department of Developmental and Comparative Psychology, Max Planck Institute for Evolutionary Anthropology, Leipzig, Germany; Emory University, United States of America

## Abstract

People often act on behalf of others. They do so without immediate personal gain, at cost to themselves, and even toward unfamiliar individuals. Many researchers have claimed that such altruism emanates from a species-unique psychology not found in humans' closest living evolutionary relatives, such as the chimpanzee. In favor of this view, the few experimental studies on altruism in chimpanzees have produced mostly negative results. In contrast, we report experimental evidence that chimpanzees perform basic forms of helping in the absence of rewards spontaneously and repeatedly toward humans and conspecifics. In two comparative studies, semi–free ranging chimpanzees helped an unfamiliar human to the same degree as did human infants, irrespective of being rewarded (experiment 1) or whether the helping was costly (experiment 2). In a third study, chimpanzees helped an unrelated conspecific gain access to food in a novel situation that required subjects to use a newly acquired skill on behalf of another individual. These results indicate that chimpanzees share crucial aspects of altruism with humans, suggesting that the roots of human altruism may go deeper than previous experimental evidence suggested.

## Author Summary

Debates about altruism are often based on the assumption that it is either unique to humans or else the human version differs from that of other animals in important ways. Thus, only humans are supposed to act on behalf of others, even toward genetically unrelated individuals, without personal gain, at a cost to themselves. Studies investigating such behaviors in nonhuman primates, especially our close relative the chimpanzee, form an important contribution to this debate. Here we present experimental evidence that chimpanzees act altruistically toward genetically unrelated conspecifics. In addition, in two comparative experiments, we found that both chimpanzees and human infants helped altruistically, regardless of any expectation of reward, even when some effort was required, and even when the recipient was an unfamiliar individual—all features previously thought to be unique to humans. The evolutionary roots of human altruism may thus go deeper than previously thought, reaching as far back as the last common ancestor of humans and chimpanzees.

## Abstract 3

Experimental evidence reveals that chimpanzees will help other unrelated humans and conspecifics without a reward, showing that they share crucial aspects of altruism with humans.

## Introduction

Individuals of many primate species perform behaviors that benefit other individuals. In particular, chimpanzees engage in behaviors such as food sharing, grooming, consolation, or coalition formation [1–6]. Most of these instances, however, can likely be explained by direct or indirect benefits for the actor through mechanisms such as mutualism or kin selection [7–8]. Humans, in contrast, occasionally help other individuals, even strangers, without immediate benefit for themselves, encompassing situations that make reciprocation unlikely [9–10].

Many researchers have claimed that such altruism is unique to humans, emanating from a species-unique psychology [10–12]. More specifically, chimpanzees (one of human's two closest living primate relatives) are often conceived as lacking the propensity to act altruistically. Only humans are supposed to develop altruistic behaviors during ontogeny, whereas chimpanzees might only be guided by self-interest. In favor of this view, in two recent experiments [12,13], chimpanzees showed no other-regarding tendencies when they could deliver food to another chimpanzee. Chimpanzees did not seem to care about the welfare of a conspecific in this food-retrieval context.

In contrast, observational studies have provided many examples of other-regarding, possibly altruistic behaviors [1–6,14]. In support of these observations, a recent experimental study [15] showed that human-raised chimpanzees provided unrewarded helping behaviors toward their human caregiver by fetching objects she was unsuccessfully reaching for, providing the first experimental evidence for altruistic helping in chimpanzees.

Current research thus suggests the possibility that chimpanzees are able and willing to help, but they display this behavior only in very restricted contexts. First, chimpanzees might help only a familiar human caregiver with whom they maintained a close relationship, based on a rearing history in which compliant behavior in other contexts had been reinforced [15]. Second, chimpanzees might help others only if costs are low, whereas humans display much more costly helping. In all experimental studies thus far, chimpanzees did not have to put much effort (energetic costs) into helping by pulling a mechanism or picking up an object. Third, it is still unclear from experiments whether chimpanzees will help another chimpanzee [16]. In previous experiments chimpanzees did not help when the beneficiary was a conspecific. It is possible that helping among chimpanzees is limited, because their relationships are often characterized by competition, especially over monopolizable food [17,18], whereas this constraint is lifted during interactions with humans who deliver food rather than compete over it. Alternatively, in these studies, chimpanzees might not have helped another chimpanzee because they were preoccupied with retrieving food for themselves, and the recipient did nothing to indicate any need for help. The current set of experiments was designed to address these issues.

## Results and Discussion

### Experiment 1

In the first experiment, we compared the helpfulness of chimpanzees and human infants toward unfamiliar individuals. Subjects were 36 semi–free ranging chimpanzees born in the wild who were tested by a human with whom they had virtually no prior interaction (no training, no feeding, no previous testing). In addition, 36 18-mo-old human infants (same age as in [15]) were tested by an unfamiliar adult in a similar context, allowing a direct quantitative comparison between species. The general idea of the testing situation was that an object was placed out of reach for the recipient, but within reach of the subject. Helping consisted of handing the object to the recipient. To assess the motivations underlying helping, we varied whether the recipient made an unsuccessful attempt to get the object (reaching versus no reaching) and whether he rewarded the subjects in exchange for the object (reward versus no reward). If subjects are responsive to the other's goal, they should hand the object more often in reaching than in no-reaching conditions. If they are primarily interested in their own immediate benefit, they should help more often in reward than in no-reward conditions.

**Figure 1:**
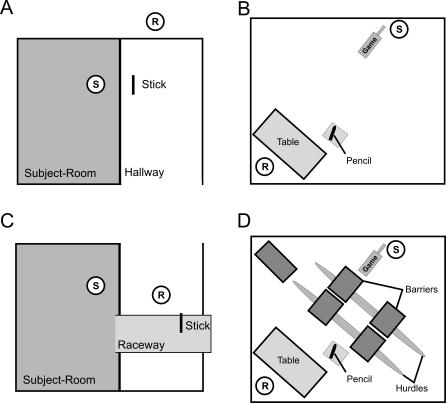
Test Areas and Setup for Chimpanzees and Human Children Experiment 1 (A, B) and experiment 2 (C, D) are shown. S indicates the subject and R indicates the recipient. The target object (chimpanzees: stick; children: pencil) was placed out of reach for the recipient but was accessible by the subject. The measured target behavior was whether the subject helped by picking up the object and handing it to the recipient.

In the chimpanzee experiment, after experimenter 1 and 2 (E1 and E2) struggled over a wooden stick to highlight its value for E1, E2 placed the stick in the hallway (Figure 1A, Video S1). Then, according to the condition, E1 would either outstretch his arm through the bars towards the stick (Reaching) or look at the object but not reach for it (No Reaching). In addition, he would either hold a piece of banana in view of the subject, which he gave them in exchange for the stick (Reward) or not have any food available (No Reward). The same basic procedure was used to test infants' helping. As a reward for the infants, we used toy cubes that were needed to play a highly motivating novel game. The helping scenario was as follows: E1 sat at the desk, using a pen to write a letter. E2 walked toward her, snatched the pen out of her hand, and put it on a stool in front of the desk out of E1′s reach (Figure 1B, Video S2). As with the chimpanzees, E1 then performed the behaviors according to the four conditions. Each subject was individually administered ten consecutive trials in one of four between-subject conditions (Reach–Reward; Reach–No Reward; No Reach–Reward; No Reach–No Reward). Thus, in each of the four conditions, nine chimpanzees and nine human infants were tested for a comparison of 72 subjects total. Each trial lasted up to 60 s: During the first 30 s, E1 vocalized and focused on the object, whereas in the remaining 30 s, E1 in addition called the subject's name and alternated gaze between the target object and the subject.

**Figure 2:**
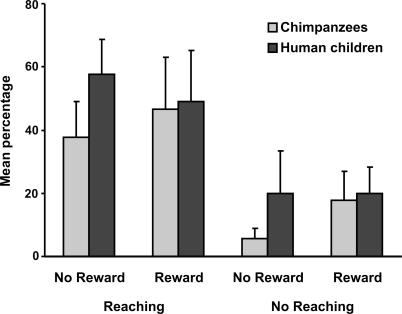
Experiment 1 Mean percentage of trials with target behavior (handing the out-of-reach object to the recipient) as a function of species and condition. Error bars represent the standard error of the mean (SEM). Each subject participated in one of the four conditions in a between-subject comparison.

We conducted a four-way mixed-models analysis of variance on the mean percentage of trials with helping, entering species, reach, and reward as between-subject and 1st versus 2nd half of session as within-subject factor. As displayed in Figure 2, helping occurred more often in reaching than in no-reaching conditions [*F* (1,64) = 14.52, *p* < 0.001], independently of species and reward (no main effects, no interactions). Pairwise comparisons using independent sample *t*-tests confirmed that reaching was the only significant factor, also when analyzed separately by species. On an individual level, 12 of 18 chimpanzees and 16 of 18 infants tested in reaching conditions helped at least once. For infants as well as for chimpanzees, the determining factor as to whether help was provided was whether the experimenter unsuccessfully attempted to retrieve the object. This indicates that subjects were motivated to help the experimenter with his/her unachieved goal (seeing the other succeed might even be intrinsically motivating for them), but did not aim at retrieving a material reward for themselves. Rewarding their helping was unnecessary and did not even raise the rate of helping in either case.

The only species difference found was that the helping of human infants was faster. Differences in reaction times should obviously be interpreted with caution because of the dissimilar locomotor skills and room setups (chimpanzees were more mobile and occasionally climbed up the experimental room and then returned for helping). However, it is of potential interest that the human children helped more often during the first 30-s phase in which the experimenter focused on the object (mean *M* = 77% of their helping acts; standard deviation *SD* = 27), in contrast to chimpanzees with *M* = 47%, *SD* = 42, *F* (1,40) = 8.05, *p* < .01 (no effect of condition). Thus, the great majority of infants' helping acts occurred before the experimenter addressed the subject, whereas chimpanzees needed these additional cues about half of the time.

Given the fact that children and even chimpanzees often displayed unrewarded acts of helping that did not significantly decrease over time, we were wondering if (1) they would continue to help when the costs of helping are slightly raised—namely, when they have to locomote some distance to retrieve the object for the other—and (2) whether we could replicate that rewarding was not necessary to have subjects help reliably across trials.

### Experiment 2

We re-tested all subjects that had helped at least once in the previous experiment (18 chimpanzees and 22 infants). The experimental setup was the same as in experiment 1, only that subjects had to put more physical effort in retrieving the object for the other. Chimpanzees had to climb up into a raceway 2.5 m above the hallway, and infants had to surmount an array of obstacles (Figure 1C, 1D, Video S3, S4). Subjects were randomly assigned to one of two conditions (reaching or no reaching) and again tested in ten consecutive trials of 60 s each. To replicate the finding that rewards are not necessary for helping, no rewards were offered in either condition.

**Figure 3:**
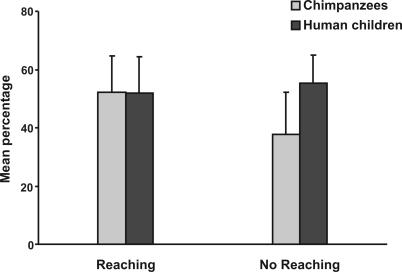
Experiment 2 Mean percentage of trials with target behavior (handing the out-of-reach object to the recipient) as a function of species and condition. Error bars represent SEM. Each subject participated in one of the two conditions in a between-subject comparison.

As shown in Figure 3, there was no difference between conditions in either species. A three-way mixed model analysis of variance on the mean percentage of trials characterized by helping, with species and condition as between-subject and 1st versus 2nd half of the test session as within-subject factor, revealed no significant main effects or interactions. This was confirmed by pairwise post-hoc comparisons using *t*-tests. Thus, chimpanzees and human infants again did not differ in the total amount of helping acts; but this time, there was also no difference between reaching and no reaching conditions. This was likely due to a carry-over effect from experiment 1 in which subjects had possibly learned that the experimenter wanted the object: The number of helping acts was highly correlated between studies: *r*(*N* = 40) = 0.68, *p* < 0.001. This correlation was apparent also when analyzed separately by species (chimpanzees: *r* (*N* = 18) = 0.79, *p* < 0.001; infants: *r* (*N* = 18) = 0.57, *p* < 0.01). Correlations held when the number of rewards received in experiment 1 was controlled. Thus, subjects who had helped more during the first experiment continued to help more in the second one, independently of whether the experimenter was now reaching for the object or not. With regard to latencies, infants again helped more often during a first 30-s phase than chimpanzees who appeared to require additional cues in order to help, with no effect of condition (infants: *M* = 67% of helping acts, *SD* = 29; chimpanzees: *M* = 33% of helping acts, *SD* = 26; *F*(1,30) = 11.39, *p* < 0.01). In sum, the second experiment demonstrated that (1) helping was sustained even if the costs were raised, and (2) replicated the finding that rewarding was not necessary to elicit helping behaviors in either species.

Taken together, experiments 1 and 2 underscore the claim that altruistic motivations are already apparent early in human ontogeny, requiring little socialization [15,19–21]. Moreover, altruistic motivations to help others do not seem to be unique to humans. Also, chimpanzees act on the behalf of others—even if this is an unfamiliar human and even if over repeated trials, they do not receive any immediate material benefit in return for their effort.

Despite the fact that subjects helped without prior or current rewarding by the same individual in a different situation, or a different individual in the same situation, it is conceivable that infants and chimpanzees had been rewarded in the past for the general behavior of handing over objects to humans. Therefore, an even more stringent test might be one with a novel task with no potential reward history at all. Human infants help in such novel tasks [15], but there is as yet no such experimental evidence with chimpanzees. Moreover, in the first two experiments reported above, chimpanzees helped a human, not a conspecific. Studies using human–chimpanzee interaction can reveal what behaviors chimpanzees are, in principle, capable of performing, but the crucial test-case for social behaviors is one in which they interact with a conspecific. Would chimpanzees also help other chimpanzees? Previous experimental results have yielded negative results [12,13]. But in these studies, subjects were preoccupied with attaining food for themselves, and the recipient was not actively trying to solve a problem. The test was thus more a test of generosity than of instrumental helping (also called “targeted helping” [22]). Therefore, we created a novel situation in which a chimpanzee individual would actively struggle with a problem, namely, trying to open a door to gain access to food, and the subject would then have the opportunity to help.

### Experiment 3

**Figure 4:**
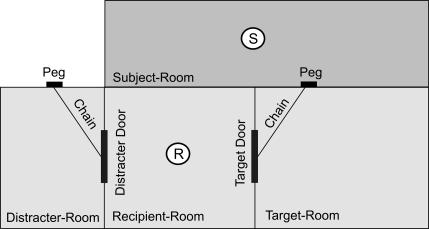
Test Area and Setup in Experiment 3 Both the target and the distracter door were held shut by chains. The recipient (R) could not access either chain, but the subject (S) could release the chain of the target door. In the experimental condition, food was placed in the target room, so that the recipient would try to open the target door and the subject could help by releasing the target chain. In the control condition, food was placed in the distracter room, so that the recipient would try to open the distracter door. In this situation, it was irrelevant (with respect to the recipient's attempt to open the distracter door) whether the subject released the target chain. The target measure in both conditions was whether the subject released the target chain.

Chimpanzees were faced with the problem that a door leading to a room with food was fixed with a chain (Figure 4, Video S5). A chain stretched from the target door to the subject room in which the subject was placed. The chain was attached to the bars with a peg, accessible only by the subject and not the recipient. Only if the subject released this chain from their room could the recipient enter. We tested nine chimpanzees from Ngamba Island who, during an individual pretest, had been skillful in manipulating the door mechanism (Protocol S1).

In the experimental condition, food was placed in the target room so that the recipient would try to open the target door and the subject could potentially help by releasing the chain. In a control condition, the food was not placed in the target room, but was placed instead behind a distracter door on the opposite side that could not be opened by either chimpanzee. If subjects were sensitive to the recipient's goal, they should produce the target behavior of releasing the chain more often in the experimental condition (when the recipient is trying to open the target door) than in the control condition (when the recipient ignores the target door).

Each subject performed both conditions of five consecutive trials each for a within-subject comparison. Three different chimpanzees served as recipients, and they were always the same for each subject in both conditions. Importantly, all were genetically unrelated group members, and the roles of recipient and subject were never reversed within a pair to exclude the possibility of simple short-term reciprocation in the same situation.

**Figure 5:**
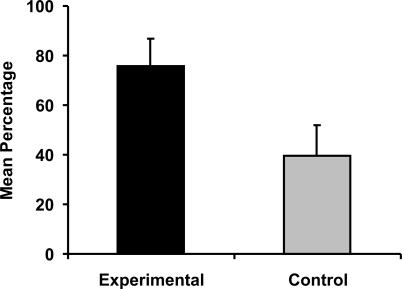
Experiment 3 Mean percentage of trials with target behavior (releasing the target chain) by condition. Error bars represent SEM. Each subject was tested in both conditions in a within-subject comparison.

For each individual, we computed a mean score of target behaviors across the five trials of each condition. Preliminary inspection of the data indicated that it was irrelevant which condition was presented first. Because we had a directional hypothesis based upon experiments 1 and 2, one-tailed tests were used. As shown in Figure 5, chimpanzees released the chain more often in the experimental than in the control condition, paired sample *t*-test, *t*(8) = 2.29, *p* = 0.025. The same result was obtained using exact nonparametric statistics, Wilcoxon matched paired test, *T^+^* = 38, *N* = 9, *p* = 0.031. In addition, the frequency of target behaviors increased over time in the experimental condition from 67% to 89% in the first two to the last two trials of a session and decreased in the control condition from 56% to 39% of the first to the last two trials, respectively (time *x* condition interaction, *F* (1,8) = 12.25, *p* < 0.01). This result suggests that subjects over time discriminated better whether help was needed or not. On an individual level, eight of nine subjects helped over multiple experimental trials, seven of them more often in the experimental than in the control condition. Analysis of latencies revealed that subjects released the chain earlier in the experimental (*M* = 20 s, *SD* = 6 s) than in the control condition (*M* = 32 s, *SD* = 5 s; *t*(5) = −2.93, *p* = 0.03).

With regard to the behavior of the recipient, we could not program the chimpanzee recipient in a similar fashion as in experiments 1 and 2 with a human recipient performing predetermined behaviors. Therefore, we assessed whether the recipient actually acted according to condition, i.e., oriented toward the target door in the experimental and toward the distracter door in the control condition. In experimental trials, recipients always positioned themselves in front of the target door. In 47 percent of control trials, however, they did not at all times remain in front of the distracter door, but approached the target door, perhaps explaining why subjects occasionally released the chain in control trials. In fact, we found that the subject's behavior was associated with that of the recipient: Across both conditions, subjects were more likely to release the chain if the recipient approached the target door than when he remained solely at the distracter door *r* (*N* = 83) = 0.37, *p* < 0.01. This analysis corroborates the finding that subjects were attentive to the recipient's attempts. They were more likely to release the chain if the recipient was unsuccessfully trying to enter through that door. The mere presence of a social partner or food cannot explain these results because that did not vary between conditions. Also, the apparatus by itself was unlikely to elicit the subject's release of the chain, as additional baseline conditions showed (Protocol S1). Importantly, we also observed no begging or harassment by the subjects after they helped, ruling out the possibility that they opened the door only to have the recipients access the food and then try to coax it from them.

### General Discussion

Taken together, the current results indicate that the altruistic tendency seen in early human ontogeny did not evolve in humans de novo. The roots of human altruism may go deeper than previously thought, reaching as far back as the last common ancestor of humans and chimpanzees. Undoubtedly, humans are exceptional with respect to the breadth in which they help, displaying helping across diverse contexts already early in infancy [15,19,21]. But, as demonstrated here, humans are not unique, because chimpanzees have the capacity to use a newly acquired skill to help a conspecific as well. This helping occurs spontaneously and repeatedly, even in a novel situation when no reward is expected and no previous rewarding could have trained them to act accordingly.

Observational studies of behaviors such as food sharing or consolation in the wild provide ecological validation to the current finding that altruistic behaviors are within the behavioral repertoire of chimpanzees. However, we know of no systematic observational study that has specifically addressed altruism in the form of instrumental help as investigated in the current experiments (helping others who fail to achieve their goal). Perhaps importantly, our experimental setup removed possible constraints such as competition over food, constraints that are prevalent in the wild and might thus often preclude altruistic behaviors.

A crucial question for future research is to determine the proximate mechanisms by which these types of helping behaviors in chimpanzees are maintained as evolutionarily stable strategies. Do chimpanzees engage in reciprocal altruism, repaying an incurred cost in the future, possibly even in a different currency [2,4,23–26]? Do they punish nonaltruistic individuals [9,27–28]? To what extent are these helping behaviors driven by empathy with the emotional states of the other, as it has been shown in the case of human infants and adults [6,20]? It is possible that one crucial difference between modern humans and their ancestors lies in the mechanisms that amplified the basic form of altruism common to humans and chimpanzees. Human cultural groups might have created unique social mechanisms to preserve and foster altruism, such as sanctioning selfish behavior and internalizing social norms [11,28,29]. But they cultivated rather than implanted the propensity to act altruistically in the human psyche.

## Materials and Methods

### Experiment 1: Participants.

We tested 36 semi–free ranging chimpanzees living at the Ngamba Island Chimpanzee Sanctuary in Uganda (21 females and 15 males aged 3 to 20 y, *M* = 10 y). All of the chimpanzees were born in the wild, were unrelated, were orphans as a result of the illegal trade in chimpanzee bushmeat, and were confiscated from poachers. During the day, the chimpanzees were released to range freely in the 39 hectares of tropical forest on the island. In the evening, the chimpanzees returned to eat food provided by caregivers and slept in a large holding facility (4 m high and approximately 140 m^2^) consisting of seven rooms (3 × 5m) with interconnecting raceways. Therefore, subjects could be tested in their indoor enclosure before being released into the forest each day. The subjects were never food deprived and water was available at all times throughout the tests. Subjects could choose to stop participating at any time. Chimpanzees were mostly naive to empirical testing. One third of chimpanzees had participated in a social learning study 3 y prior to our experiment [30]. Most importantly, none of the subjects had previously been fed or tested by the first experimenter (BH).

In addition, 36 children of 18 mo of age participated (17.2–18.5 mo). This age-group was selected because it represents the earliest age at which robust instrumental helping has been demonstrated so far [15]. Children were all native German speakers and came from heterogeneous socioeconomic backgrounds. None of the children had previously participated in experiments on helping.

### Experiment 1: Procedure with chimpanzees.

The test area consisted of the subject room (3 × 5 m), a hallway, and the outside area of the holding facility, all segregated through metal bars. The first experimenter (E1) always sat outside the facility in front of a door leading to the hallway. In each trial, experimenter 2 (E2) approached E1 and started to struggle with him over a wooden stick (16 cm long; 4 cm in diameter). This was done to highlight its value for E1 in a context familiar to chimpanzees (competition over objects). After successfully pulling the stick out of E1′s hand, E2 entered the hallway and shut the door. E2 put a piece of food in the hallway to position the chimpanzee at the starting location, placed the stick on the ground, and left toward the end of the hallway. The chimpanzees could easily reach the stick from the chimpanzee room through a hole in the bars, but this was impossible for E1 because he was located behind the door.

Once the stick was in place and the subject had taken the food, E1 would perform the following behaviors according to the four conditions (Reach–Reward; Reach–No Reward; No Reach–Reward; No Reach–No Reward): He either outstretched his arm toward the stick through the bars of the door and vocalized (Reaching) or sat in front of the door, looking at the object, but not reaching for it (No Reaching). In addition, he would either hold a piece of banana in view of the subject which he gave them in exchange for the stick (Reward) or did not have any food available (No Reward).

In all conditions, each trial lasted up to 60 s, divided up into two phases: During the first 30 s, E1 vocalized and focused primarily on the object. If the chimpanzees did not hand him the stick during this period, he then became louder by banging at the door, calling the subject's name and gaze-alternating between the stick and the subject for an additional 30 s. Trials ended after the subject handed the stick or after the allotted maximum time of 60 s. After the trial, E2 either picked up the stick from the hallway (if subjects had not retrieved it) or snatched it out of E1′s hand (if subjects had helped).

At the beginning and the end of each session, a baseline was administered with the stick lying in front of the subject in the hallway but no other person present. Subjects very rarely took the stick and put it back outside the cage when no one else was there. Taken together, subjects took the stick in 39 of 72 baseline trials (26 pre- and 13 posttest trials) and placed it back outside only five times.

### Experiment 1: Procedure with children.

The same basic procedure was used to test children's helping behavior. Again, 36 subjects were individually tested in the four conditions with ten consecutive trials each, all performed within one testing session of approximately 20 min. Instead of food, we used toy cubes which were needed to play a novel game: Upon throwing the cube through an opening, it would slide down a transparent tube into a box and create a jingle sound. This game was introduced during the warm-up phase. Children were highly motivated to play the game throughout the test (e.g., when E1 offered them the toy cube as a reward, they threw it into the box in 57 of 58 instances).

Children were tested in a room of 4.5 × 4 m, accompanied by a parent who sat in a corner of the room and remained passive during the session. The room contained a desk, a stool located 0.5 min front of the desk, and the tube game placed at the wall opposite to the desk. E1 sat at the desk and E2 operated a camera in the corner of the room. The helping scenario was as follows: E1 sat at the desk, using a pen to write a letter. E2 walked towards her, snatched the pen out of her hand, used it shortly on her notepad and then put it on a stool out in front of the desk out of E1′s reach. To locate the child in the starting position, she then placed a toy cube on the tube apparatus. After the child had thrown the cube down the tube, E1 performed the behaviors according to the four conditions: Reaching for the pen by bending over the desk or looking at it (Reaching versus No Reaching), either with a cube as a potential reward held in her hand or without (Reward versus No Reward). Again, in all conditions each of the ten consecutive trials lasted up to 60 s, split in two 30-s phases: (1) focusing on the object and vocalizing, and (2) addressing child through gaze-alternation and name calling.

If the child handed the pencil to E1, she used it on her sheet of paper and continued with the next trial. E1 never praised the child. In Reward conditions, children received a block in exchange for the pencil. Trials ended when the children handed the pen over or after a maximum of 60 s. If the child did not bring the pencil after 60 s, E2 walked to the stool, took the pencil for a quick note on her pad and placed it on the desk where E1 could reach it for the next trial. To prevent frustration, a short play period was interspersed if children did not bring the object for three consecutive trials.

### Experiment 2. Design and procedure.

Chimpanzees were tested 1 mo and children on average 8 d after experiment 1. The major procedural difference to experiment 1 was that in the chimpanzee experiment, the stick was not placed next to the subject's starting position on the ground, but in a raceway 2.5 m above, where chimpanzees had to climb in order to retrieve it. Thus, after E2 had pulled the stick out of E1′s hand, she placed a banana on the ground to get the chimpanzee in the starting position, put the stick up in the raceway and left. After that, E1 entered the hallway and stood underneath the raceway, either reaching or not reaching for the stick (contingent upon condition). Subjects were tested in ten consecutive trials of up to 60 s each. During the first 30 s, E1 would look at the chimpanzee, and during the last 30 s, start to alternate gaze and increase his effort to retrieve the stick through vocalizing, hand clapping, and name calling. Sessions lasted approximately 20 min. At the beginning and the end of each session, two baseline trials were administered: In a food-for-self baseline, we put a piece of banana up in the raceway to ensure that the chimpanzees would climb up to the raceway at all, which they always did. In a stick-baseline, we put the stick in the raceway with no other person present. Chimpanzees entered the raceway in 14 of 18 trials at the beginning and 10 of 18 trials at the end of the sessions, but transferred the stick into the hallway only four times altogether (two times before and two times after the session). Thus, without anybody else present, chimpanzees virtually never performed the target behavior.

In the children experiment, the same room setup as in the first child experiment was used, but subjects had to surmount an obstacle of two soft toy snakes lying across the room, often requiring the children to stabilize themselves with one hand at boxes aligned to the side, which served as a kind of handrail. Before testing, an obstacle control condition was administered to assure that the children are not afraid of the obstacle or locomotorically impaired. The child stood in the starting position while E1 was on the other end of the obstacle, encouraging the child to walk across the obstacles to take an attractive toy she was holding. This was repeated up to three times until the children passed the obstacles once. All children met this criterion. Again, infants were tested in ten consecutive trials of up to 60 s each, divided up in two 30-s phases (focusing on object; addressing the child).

### Experiment 3. Design and procedure.

All subjects were tested in both the experimental and the control condition on two separate days, spaced 1 to 2 d apart. Four subjects started with the experimental condition and the other five subjects with the control condition. Each condition consisted of five consecutive trials, administered during a single session of approximately 20 to 30 min. At the beginning and the end of each session, a baseline condition was conducted in which neither a recipient nor food were present.

In all conditions, a chain stretched from the target door to the subject room in which the subject was placed. The chain was attached to the bars with a peg, accessible only by the subject and not the recipient. The subject room was mostly empty, containing only some straw and a rope attached to the bars, with which the subject could play during the trials. In the experimental and the control condition, a second chimpanzee was placed in the recipient room. In the baseline, no second chimpanzee was present. Trials ended after the subject released the peg or after the allotted maximum time of 60 s.

In the experimental condition, E1 first placed food (a piece of banana or watermelon) on the floor in the target room behind a tire, visible to the recipient but invisible to the subject. E1 then attached the chain with a peg between the bars of the helper room. At the same time, E2 and E3 distracted the subject by giving food in the corner of the helper room so that E1 could safely attach the peg.

In the control condition, the same procedure was used, with the crucial difference that food was placed not in the target room, but behind the distracter door on the opposite side. The distracter door could not be opened by either the recipient or the helper.

It was of course critical that the recipient would constantly make attempts to enter through the respective doors. If recipients did not orient towards the appropriate door, the trial was repeated. This was necessary only six out of 90 times (one experimental and five control trials). To ensure that recipients would continue to orient toward the distracter door in control trials, we interspersed motivation trials in which the distracter door could be slid open by the recipient and the food could therefore be accessed. Correspondingly, if recipients did not receive help for two consecutive experimental trials, we included a motivation trial in which food was placed in the target room and the door could be opened by the recipient.

The procedure of the baseline conditions was similar to that of the other conditions, only that no food was placed in either location and no recipient was present.

## Supporting Information

Protocol S1 Supporting Materials and Methods (54 KB DOC)

Video S1 Experiment 1 Chimpanzee in condition Reaching/No Reward.
(8.9 MB MPG)

Video S2 Experiment 1 Child in condition Reaching/No Reward.
(9.5 MB MPG)

Video S3 Experiment 2 Chimpanzee in condition No Reaching.
(6.9 MB MPG)

Video S4 Experiment 2 Child in condition Reaching.
(7.3 MB MPG)

Video S5 Experiment 3 Chimpanzee helps in experimental condition.
(4.4 MB MPG)

## Abbreviations:

E1: experimenter 1
