# Supplementary material for: Spontaneous Altruism by Chimpanzees and Young Children
Source: PLoS Biol. 2007 Jun 26;5(7):e184. doi: 10.1371/journal.pbio.0050184 (PMC1896184; doi:10.1371/journal.pbio.0050184)
Supplement: Supplementary file 1 [file pbio.0050184.sd001.doc]

**Supplementary Information**

**Spontaneous Altruism by Chimpanzees and Young Children**

**F. Warneken, B. Hare, A. P. Melis, D. Hanus & M. Tomasello**

**Experiment 1**

***Coding***

All coding was done from videotape. To assess inter-rater agreement, 20% of trials were independently coded by a second person. The chimpanzee study was coded by a research assistant and co-coded by the first author; the children study was coded by the first author and co-coded by a different research assistant. We assessed whether subjects did or did not help during a given trial (yes/no), defined as handing the object to the experimenter so that he could successfully retrieve it (chimpanzees: κ = .96; children: κ = 1.0) and the corresponding latencies (highly correlated between coders: chimpanzees: *r*(N = 17) = 1.0, *p* < .001; children: *r*(N = 19) = .99, *p* < .01).

**Experiment 2**

***Coding***

Ratings and raters were the same as in the previous study, once again resulting in high inter-observer agreement: Target behavior yes/no with κ = .87 and 1.0 for chimpanzees and children, respectively. Latencies of target behavior with *r*(N = 21) = .98, *p* < .01 for children and *r*(N = 10) = 1.0, *p* < .001 for chimpanzees.

**Experiment 3**

***Participants***

The final sample consisted of 9 chimpanzees from Ngamba Island who had been able to operate the door mechanism during the pretest which is described below (6 additional chimpanzees failed the pretest). Three chimpanzees served as recipients; one of them had been a subject in a previous session of the experiment (a male) and the remaining two were never subjects (both females).

***Setup and Materials***

*Pretest 1: Introduction to door mechanism*

Before subjects could be used for the helping experiment, they had to demonstrate that they understood the door mechanism. Therefore, we performed individual pretests in which subjects were faced with the problem that they could not enter a room with food because a chain was blocking the sliding door leading to it. To solve the problem, they first had to release the chain and then enter through the door.

The subject could freely move through rooms 1 and 2, but the food was placed in the room 3 (see Supplementary Figure 1). The sliding door between rooms 2 and 3 was blocked by a chain which was hooked up with a peg in the bars of room 1. Thus, the subjects had to first release the chain in room 1 by twisting the peg and then open the door from room 2 in order to enter room 3. The approximately 2.5 m long chain was attached to a sliding door and was hooked up on the other end with a peg horizontally between bars (bar width 10 cm). The peg had to be twisted from its horizontal to a vertical position, so that it would fit through the space between the bars.

Supplementary Figure 1: Test area as used for individual pretest in experiment 3 (top view).

We wanted to ensure that the subjects did actually understand that the chain needed to be released *in order to* enter through the door, rather than as two independent events occurring accidentally. Therefore, we set as criterion that subjects (1) had to release the chain within 60 seconds and enter through the door within 20 seconds after its removal and (2) do so in two consecutive trials. In a first day of pilot testing, several subjects failed to twist the peg in three trials when they had to enter another room in order to release the chain and then return to the door, also if a human experimenter demonstrated the mechanism. Therefore, on a second day, the setup was made easier for these subjects in that they could reach the chain and the peg directly while they were sitting in room 2 in front of the door. Four of the 9 final subjects received the easier condition first. In a last pilot session with the original setup in which they had to release the chain from room 1 and slide the door open from room 2, all 9 subjects of the final sample reached criterion within only two trials.

*Pretest 2: Inhibition Test*

After the introduction to the mechanism it was possible that the subjects would release the chain irrespective of any need to do so. To offer them an alternative activity, we attached a ca. 3 m long rope to the bars of the subject room. This rope was nonfunctional, but the subjects could pull it inside their room and play with it. After introducing the rope, we ran inhibition trials with the criterion that subjects did not release the peg for 60 seconds in two consecutive trials during which no food was accessible and no other individual was present. Subjects included in the final sample reached the criterion after 2 to 4 trials.

***Coding***

A research assistant coded the sessions from videotape. Five experimental trials were not on tape, thus live coding was used instead. To assess inter-rater agreement, 20% of trials were independently coded by the first author. We coded whether the helper did or did not release the chain during the trial (target behavior, κ = 1), the corresponding latency (correlation between coders: *r*(N = 10) = 1.0, *p* < .001) and whether the helper begged for food from the recipient after helping (no instances observed by either coder). As it was essential that the recipient acted according to condition, i.e. was oriented towards the target door in the experimental condition and *not* to the target door in the control condition, we assessed which of the two doors they approached during the trial (κ = 1 for the distractor and κ = .88 for the target door).

***Supplementary Results***

For each individual we computed a mean score of target behaviors across trials of each condition. An analysis of variance with condition (Experimental, Control, Baseline) as within-subject factor and the mean percentage of trials with target behavior as dependent variable yielded significant results, *F* (2,16) = 5.06, *p* = .02 (two-tailed). Subsequent paired sample t-tests (one-tailed) revealed that subjects released the chain more often in the experimental than in the control condition, *t* (8) = 2.29, *p* = .025. In the baseline condition, subjects released the peg in *M* = 39 percent (*SD* = 41) of trials (averaged across the 4 trials of the two session). Thus, also in the baseline condition subjects were less likely to release the chain than in the experimental condition, *t* (8) = 2.43, *p* = .02.
